# Advice and Guidance in Mental Health: A Transformational Approach

**DOI:** 10.1192/bjo.2024.500

**Published:** 2024-08-01

**Authors:** Viviane Nzouonta Ngwompo, Paul Maddock, Rebecca Harris

**Affiliations:** Avon and Wiltshire Mental Health Partnership NHS Trust, Bath, United Kingdom

## Abstract

**Aims:**

- To strengthen shared decision making between psychiatrists and general practitioners (GPs) while avoiding needless outpatient activity.
- To promote a seamless partnership between GPs and psychiatrists that will improve efficiency and effectiveness for better patient health outcomes.
- To improve patient journey whilst responding to operational pressures.
- To test GPs engagement and satisfaction.

**Methods:**

Following GPs engagement sessions, a 12 weeks pilot was conducted with the Bath and North East Somerset (BaNES) Primary Care Liaison Service (PCLS) and the 6 Primary Care Networks (PCNs) in BaNES. 22 GP surgeries were allowed access to Advice and Guidance (A&G) system using a digital platform. The pilot ran from 3rd April to 25th June 2023, focussing on answering non-urgent queries related to: psychotropic medications, mental health presentations, and the wider mental health system signposting and awareness.

One Consultant Psychiatrist and One Associate Specialist in Psychiatry were involved. The asynchronous system (eOpinion) with an expected response time of 3–5 working days was used.

To allay any governance risks and to act as a backup should the A&G system process fail to record appropriately, a dual recording of the A&G given – both in the A&G digital platform and the patient electronic record was implemented. Further governance structures were built into the project to establish that actions undertaken by the psychiatrists were effective and justifiable.

**Results:**

82 requests received over the 12 weeks period.

20 out of 22 surgeries took part.

The Psychiatrists spent on average of 3.5 hours per week answering A&G requests. The administrative team spent on average one hour and three quarter per week processing A&G responses.

Although no significant impact on total referrals was noted, there was indication that demand was moving from the referral to A&G request.

All requests were responded within 2 working days.

Requests from GPs were largely appropriate with 88% resulting in advice and guidance, indicating an improved patient journey.

Minimal impact on the operational processes.

Positive feedback from GPs with 91% finding the A&G system useful or very useful. They were keen for the offer to continue.

**Conclusion:**

Effective inter-professional collaboration between GPs and psychiatrists is essential in enhancing patients' overall health outcomes and experiences. For mental health services, this transformational approach should continue to enhance the existing offer. However, we should remain mindful of the potential risk of increased workload burden in General Practices, and the implications of this new clinical model on staff based in specialist services.

